# Symptom Burden Poorly Responsive to Palliative Care Intervention and Karnofsky Predict Survival in an Acute Palliative Care Unit

**DOI:** 10.3390/cancers17101704

**Published:** 2025-05-19

**Authors:** Sebastiano Mercadante, Yasmine Grassi, Alessio Lo Cascio, Alessandra Casuccio

**Affiliations:** 1 Main Regional Center of Pain Relief and Supportive/Palliative Care, La Maddalena Cancer Center, Via San Lorenzo 312, 90146 Palermo, Italy; grassi.yasmine@lamaddalenanet.it (Y.G.); locascio.alessio@lamaddalenanet.it (A.L.C.); 2 Department of Health Promotion, Maternal and Infant Care, Internal Medicine and Medical Specialties, University of Palermo, 90127 Palermo, Italy; alessandra.casuccio@unipa.it

**Keywords:** palliative care, survival, Karnofsky, Edmonton Symptom Assessment System, cancer

## Abstract

**Background/Objective:** Survival prediction in the advanced cancer care setting plays a vital role **in** treatment planning and patients’ arrangements. The aim of this study was to examine the association of the global Edmonton Symptom Assessment System (GESAS) and Karnofsky scale (KPS) with overall survival (OS) in patients with advanced cancers admitted to an acute palliative care unit (APCU). The second aim was to assess if GESAS changes after comprehensive palliative treatment could influence OS. **Methods:** This is a prospective planned sub-analysis of advanced cancer patients. A consecutive sample of 521 patients admitted to an APCU. Patients with available survival in follow-up phone calls, having complete ESAS, and discharged alive were selected. KPS and GESAS were measured at admission and after seven days of individual comprehensive palliative care. **Results:** Two hundred forty-three of 521 screened patients were assessed according to inclusion criteria. The mean age was 67.1 years (SD 11.5), and 121 patients were male. The mean KPS was 43.5 (SD 9.3). The mean OS was 74.6 (SD 136.2) days. Significant changes in GESAS were observed after one week. Univariate linear regression analysis showed that KPS and GESAS at T0 and at T7 were correlated with OS (*p* < 0.0005; *p* = 0.020; *p* < 0.0005, respectively). At multivariate analysis, OS was correlated with KPS and GESAS at discharge (B = 3.349, 95% CI = 1.560–5.137; B = −2.430, 95% CI = −3.831–−1.029). **Discussion:** KPS and poor response to intensive treatment, maintaining high GESAS scores, can be considered predictive factors of shorter OS. Further studies should confirm whether a specialized intervention in other settings can improve OS.

## Simple Summary

Survival prediction in the advanced cancer care setting plays a vital role in treatment planning and patients’ arrangements. The aim of this study was to examine the association of the global Edmonton Symptom Assessment System (GESAS) and Karnofsky scale (KPS) with overall survival (OS) in patients with advanced cancers admitted to an acute palliative care unit (APCU). KPS and high GESAS scores after intensive treatment can be considered predictive factors of shorter OS. Further studies should confirm whether a specializedintervention in other settings can improve OS.

## 1. Introduction

Survival prediction in the advanced cancer care setting plays a vital role in treatment planning and patients’ arrangements. A common need in these patients is the accurate assessment of prognosis by their medical care team in order to drive informed decisions [1]. The Edmonton Symptom Assessment System (ESAS) is a simple, validated tool measuring ten common symptoms in patients with advanced cancer, including pain, dyspnea, anxiety, depression, poor sleep, drowsiness, weakness, nausea, poor appetite, and poor well-being. In a previous study, at the first visit to an outpatient palliative care clinic, high symptom burden, expressed by the global ESAS (GESAS), was associated with a decrease in overall survival (OS), highlighting the potential role of the ESAS as a tool in prognostication for patients with advanced cancers with a high symptom burden [2]. Another simple tool, the KPS, has been found to be a useful predictor of OS in patients attending palliative care services [3]. However, GESAS was measured only at the moment of referral, as this was a cross-sectional study and not longitudinally measured. Highly specialized interdisciplinary care units such as acute palliative care units (APCU) can provide intensive symptom management, allowing the achievement of rapid pain and symptom control [4]. The response to symptom management, with possible changes in GESAS, has not been taken into consideration, particularly in an acute palliative care unit (APCU), where comprehensive and acute palliative care treatment is rapidly effective in reducing symptom burden [4].

The aim of this study was to examine the association of GESAS and KPS with OS in patients with advanced cancer to confirm previous studies performed in other settings in a cross-sectional way in a large sample of patients admitted to the APCU. The second outcome was to assess if ESAS changes after intensive comprehensive palliative treatment could influence OS.

## 2. Methods

This is a pre-planned sub-analysis of a prospective study assessing the activities of an APCU. The study was conducted within an APCU, devoted to teaching and research, and affiliated with the University of Palermo. The unit comprises 12 beds and has been operational for over 25 years within a comprehensive cancer center. The characteristics of this unit have been described elsewhere [4]. The study was conducted according to the guidelines of the Declaration of Helsinki and approved by the provincial ethical committee of Palermo (Comitato Etico Provinciale di Palermo 1. Prot.PSCU-00 VR1 06.09.20.23, n.05/2023, 14 November 2023). Patients’ informed consent was obtained.

### 2.1. Patients

A consecutive sample of cancer patients admitted to the APCU was prospectively assessed over 13 months. Inclusion criteria were advanced cancer, adult patients, and patients discharged alive from the unit. Exclusion criteria were non-collaborative patients or those expected to die in the unit.

All patients underwent comprehensive palliative care treatment, including continuous symptom assessment and personalized therapeutic interventions based on the specific needs and characteristics of patients.

### 2.2. Data Collection

Demographic data (age, gender, primary diagnosis) and KPS were recorded at admission. Symptom burden was assessed using the Edmonton Symptom Assessment Scale (ESAS), a validated tool measuring symptom severity on a 0–10 scale, sensitive to changes produced by treatment [5]. Patients underwent specialized assessment and symptomatic treatment in the APCU. Symptom burden was calculated by the sum of ESAS intensities (GESAS) at admission (T0) and after seven days of individual comprehensive palliative care (T7). We followed the STROBE checklist for reporting observational studies.

After discharge, follow-up phone calls were performed, with the last being performed three months after completion of the study. Patients for whom survival was available in follow-up phone calls, having complete ESAS, and being discharged alive were selected. Patients with severe cognitive disturbances or who were not collaborating were excluded.

The association of GESAS and KPS with OS in patients with advanced cancer was examined to confirm previous studies performed in other settings in a cross-sectional way in a large sample of patients admitted to the APCU. Secondarily, whether ESAS changes after intensive comprehensive palliative treatment could influence OS.

### 2.3. Statistical Analysis

Continuous data are expressed as mean ± SD, and not-normally distributed variables were summarized as median (interquartile range, IQR). The paired Wilcoxon signed-rank test was used to compare pain intensity scores and symptom intensity scores over the time intervals. Linear regression analysis examined the correlation between patient characteristics (independent variables) and OS (dependent variable) in univariate and multivariate regression models.

To assess the predictive rate of different cutoffs of KPS and GESAS at T7 values, according to patient survival, this was dichotomized into two groups with respect to median value (<33 days and ≥33 days). Hence, a receiver operating characteristic (ROC) curve with calculations of area under the curve and 95% CI was constructed, and sensitivity and specificity values were calculated. Data were analyzed by IBM SPSS Software 24 version (IBM Corp., Armonk, NY, USA). All *p*-values were two-sided, and *p* ≤ 0.05 was considered statistically significant.

## 3. Results

Two hundred forty-three of 521 screened patients were assessed according to inclusion and exclusion criteria. The mean age was 67.1 years (SD 11.5), and 121 patients were male. The mean Karnofsky was 43.5 (SD 9.3). The mean OS was 74.6 (SD 136.2) days, and the median value was 33 days (interquartile range 14–74 days). Significant changes in GESAS were observed after one week (Table 1).

Univariate linear regression analysis showed that Karnofsky, GESAS at T0, and GESAS at T7 were correlated with OS (*p* < 0.0005; *p* = 0.020; *p* < 0.0005, respectively). At multivariate analysis, OS was correlated with Karnofsky level and GESAS at discharge (B = 3.265, 95% CI = 1.478–5.052; *p* < 0.0005; B = −2.766, 95% CI = −4.157–−1.376; *p* < 0.0005; respectively) (Table 2).

The median overall patient survival value was 33 (interquartile range 14–74). This value was used to dichotomize the patients into two categories: those who survived less than 33 days and those who survived 33 days or more.

**Figure 1:**
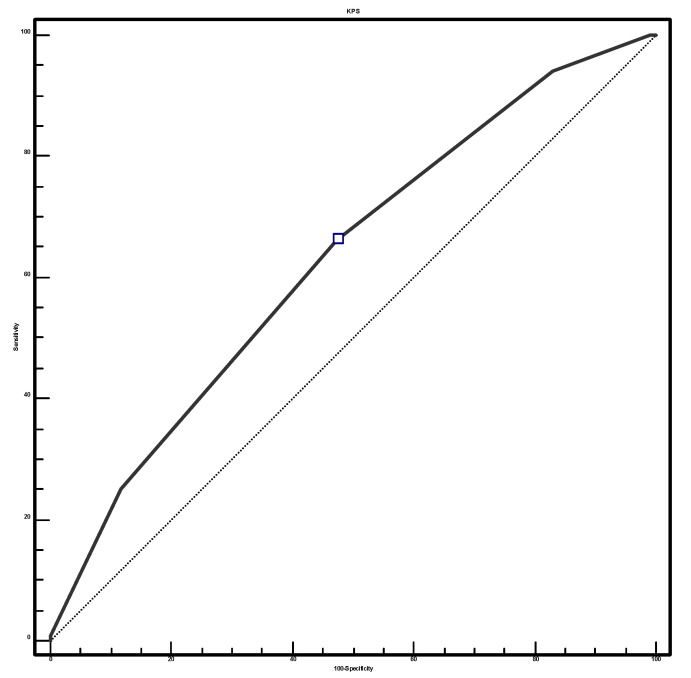
ROC curve for survival in patients with a Karnofsky value ≤ 40.

**Figure 2:**
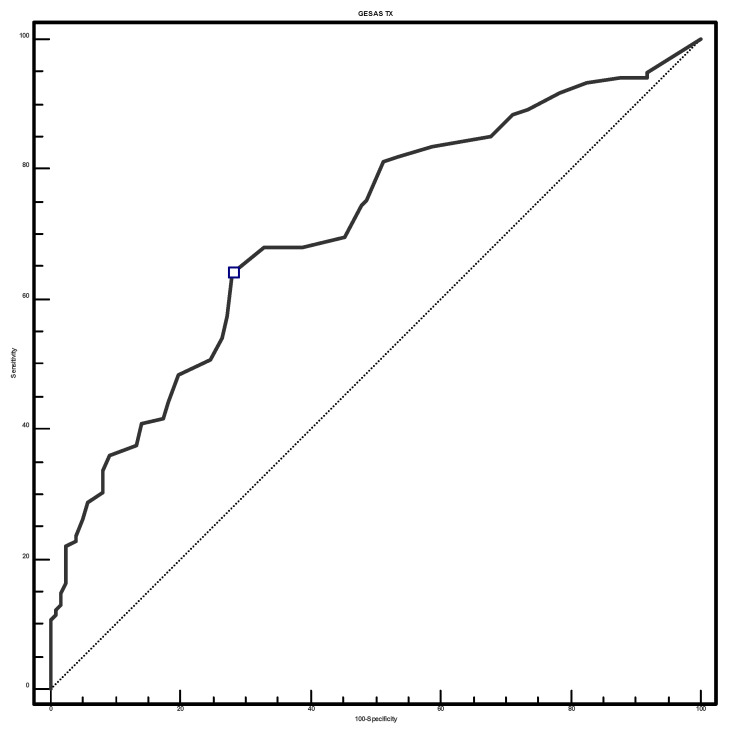
ROC curve related to GESAS at baseline.

To assess the predictive rate of different cutoffs of KPS and GESAS at T7 values according to patient groups dichotomized for overall survival, we performed a ROC (receiver operating characteristics) curve that identifies for a Karnofsky value ≤ 40 an area under the curve (AUC) of 0.629 (sensitivity 66.4% and specificity of 52.5%, *p* = 0.0004), and for GESAS at T7 > 17 an AUC of 0.707 (sensitivity 63.9% and specificity of 71.9%, *p* < 0.0005) with *p* < 0.001, and that can thus be considered a statistically significant predictor of low overall survival. (Figure 1 and Figure 2).

## 4. Discussion

Data from this study showed that a high burden of ESAS symptoms unresponsive to comprehensive palliative care treatment and a low Karnofsky were independently associated with a shorter OS. This suggests that symptom improvement achieved in an APCU may positively influence OS, while patients poorly responsive to specialized treatment, maintaining a high GESAS, will have a shorter survival.

In studies performed in different settings, KPS was a useful predictor of survival. KPS was strongly associated with the overall survival of patients with cancer attending a palliative care service [3]. In other studies in which GESAS and Karnofsky were assessed as survival predictive factors, they were found to be associated with OS. In patients with a first outpatient palliative care consultation, a higher GESAS was associated with a clinically significant decrease in OS, highlighting the potential of the ESAS as a patient-reported outcome tool in prognostication and clinical decision-making for patients with advanced cancers with a high symptom burden [2]. In a retrospective study, patients receiving first-line sunitinib therapy for metastatic renal cancer with baseline symptom burden measured by ESAS score appeared to provide prognostic information on survival [6]. In patients seen at initial oncology consultation, the severity of the ESAS total symptom burden score was found to be positively correlated with performance status. Patients with a higher symptom burden were less likely to receive systemic chemotherapy than those with fewer symptoms and had a significantly reduced OS [7]. However, in these studies, GESAS was assessed at baseline only, with no further longitudinal assessments, and performed in outpatients or single consultations, which are settings in which intensive treatment is unlikely to be performed. Indeed, one of the aims of the present study was to evaluate if an intensive intervention for symptom control in an APCU could improve survival in patients who were responsive. This means that there are patients who deteriorate and, without an intervention, may have a short survival. This is the case for a range of patients. However, there is another range of patients in which deterioration is reversible as it responds to intensive treatment in an APCU. This is consistent with studies performed with different methods and settings, which suggest that recognition and monitoring of patient-reported outcomes may contribute to improving survival after palliative care initiation [8,9,10,11]. An admission to an APCU is characterized by a rapid decrease in symptom burden in a short time, due to precise assessment and specialized palliative care intervention [12,13]. Thus, maximal efforts should be made in such intensive units, emphasizing their role in a comprehensive cancer center.

The principal limitation of this work was the single-institution study in an intensive APCU, in which referral was dictated by several clinical conditions, including pain control, symptom management, toxicity, and worsening of clinical conditions. Thus, data cannot be generalized to other settings. This was a pre-planned sub-analysis of a prospective study assessing the activities of an APCU. Therefore, data included in the original study were assessed, focusing on two parameters recently reported as negative predictive factors and excluding the many other factors reported in the literature [14]. However, all these studies lack dynamics, that is, a longitudinal assessment after an intervention. Finally, survival was not available for all patients, despite periodic phone calls, and was limited to 3 months after stopping the recruitment.

## 5. Conclusions

KPS and a high GESAS score after an effective intensive treatment can be considered predictive factors of shorter OS. Further studies should confirm whether symptom management in other palliative care settings can improve OS.

## Figures and Tables

**Table 1:** Changes in ESAS items after one week of palliative care treatment.

	Mean (SD) T0	Mean (SD) T7	*p* *
Pain	3.75 (2.7)	1.77 (1.8)	<0.0005
Dyspnea	1.32 (2.2)	0.57 (1.3)	<0.0005
Anxiety	2.84 (3.1)	1.43(2.2)	<0.0005
Depression	2.32 (3.0)	1.16(2.2)	<0.0005
Poor sleep	4.28 (3.2)	1.9 (2.6)	<0.0005
Drowsiness	2.92 (2.6)	2.1 (2.2)	<0.0005
Nausea	1.13 (2.3)	0.33 (1.2)	<0.0005
Poor appetite	3.9 (3.3)	2.1 (2.7)	<0.0005
Weakness	5.8 (2.8)	3.6 (2.7)	<0.0005
Poor well-being	5.1 (2.7)	2.8 (2.5)	<0.0005
Global ESAS	33.4 (14.3)	17.9 (13.1)	<0.0005

* Wilcoxon signed-ranks test.

**Table 2:** Univariate (Crude OR) and multivariate (Adjusted OR) linear regression analysis for the correlation among the clinical characteristics and overall survival of patients.

	Overall Survival			
	Crude OR(95% CI)	*p*-Value	Adj-OR(95% CI)	*p*-Value
Age	−1.087 (−2.58–0.413)	0.155	-	-
Sex (F vs. M)	19.987 (−14.416–54.390)	0.254	-	-
Karnofsky	3.629 (1.786–5.472)	<0.0005	3.265 (1.478–5.052)	<0.0005
Total ESAS at T0	−1.417 (−2.610–−0.223)	0.020	−0.300 (−1.554–0.955)	0.638
Total ESAS at T7	−3.006 (−4.268–1.744)	<0.0005	−2.766 (−4.157–−1.376)	<0.0005

## Data Availability

Data are available on request.
